# Supplementary material for: Association between attitudes of stigma toward mental illness and attitudes toward adoption of evidence-based practice within health care providers in Bahrain
Source: PLoS One. 2019 Dec 2;14(12):e0225738. doi: 10.1371/journal.pone.0225738 (PMC6886841; doi:10.1371/journal.pone.0225738)
Supplement: S1 Table — There are only 13 studies reported that used the 15-item OMS-HC. An overview of the participants, full scores, Cronbach’s alphas, and some of the remarks and main conclusions are highlighted. (PDF) [file pone.0225738.s001.pdf]

S1 Table. Brief literature review on 15-item OMS-HC. There are only 13 studies reported utilized 15-item OMS-HC and an overview of the participants, full score, Cronbach's alphas, some of the remarks and main conclusions are highlighted.

No.	Study	Participants	Full score	Cronbach's alphas	Remarks	Conclusions
1	**Modgill G, et al., (2014)**	Healthcare providers in Canada.<br>Two groups:<br>1. Pre-test(n=1448).<br>2. post-test (n=803).	1. Pre-test=33.4.<br>2. post-test=31.2.	Crude(everyone)=0.79.	For more information please see full text and additional file 6.	Acceptable internal consistency of new version 15-item OMS-HC and reported good in detecting a positive change in anti-stigma interventions.
2	**Knaak S, et al., (2015)**	• Assessing anti-stigma program among healthcare providers in Canada.<br>• Two versions employed:<br>1. Original version(n=94)<br>2. Borderline Personality Disorder (BPD) version(n=93).	1. Original<br>• Pre-intervention=30.35.<br>• post-intervention=29.28.<br>2. BPD<br>• Pre-intervention=37.56.<br>• post-intervention=32.83.	1. Original<br>• Pre-intervention=0.73.<br>• post-intervention=0.81.<br>2. BPD<br>• Pre-intervention=0.79.<br>• post-intervention=0.8.	Compare the stigma of mental illness and the stigma of BPD.	Providers showed higher BPD stigma than other mental illness.<br>Anti-stigma programs reduce stigma significantly in both groups.
3	**Fernandez A, et al., (2016)**	• Medical students from Malaysia.<br>• Anti-stigma lecture with:<br>1. Face-to-face intervention(n=51).<br>2. Video-based contact(n=51).	1. Face-to-face<br>• Pre-intervention=43.3.<br>• post-intervention=35.6.<br>2. Video-based contact<br>• Pre-intervention=42.5.<br>• post-intervention=34.6	Only reported the pre-treatment sub-scales= 0.5 for attitude, 0.6 for disclosure and help-seeking and 0.60 for social distance.	Assessment 1-month follow-up reported.	Coupled interventions of educational lecture and either face-to-face contact or video-based contact reduce stigma within health care providers.
4	**Petkari E (2017)**	Students of BSc Psychology program in UAE(n=26) through cine-forum interventions.	• Pre-intervention=36.56.<br>• post-intervention=34.41.	Not available	Only 18 completed the assessment.	The benefit of movie-based interventions as a learning tool for mental illness and stigma reduction in a restrictive environment for psychology students.

No.	Study	Participants	Full score	Cronbach's alphas	Remarks	Conclusions
5	**Chang S, et al., (2017)**	Healthcare students of medical(n=502) and nursing(n=500) in Singapore.	14-item OMS-HC:<br>• Total=35.7<br>• medical=34.5<br>• nursing=36.8	14-item=0.75	• First large study that validated OMS-HC in Asia.<br>• Factor analysis retained only 14-item OMS-HC.	Medical students were found to be less stigmatized than nursing.
6	**Beaulieu T, et al., (2017)**	Primary care physicians(n=111) randomized to control or anti-stigma intervention of novel skill-based approach 'The British Columbia Adult Mental Health Practice Support Program.	1. Control group:<br>• pre-test=31.65 (mean=2.11).<br>• post-test=31.05 (mean=2.07).<br>2. Intervention group:<br>• pre-test= 33.15 (mean=2.21).<br>• post-test= 31.05 (mean=2.07).	1. Control group:<br>• pre-test=0.52.<br>• post-test=0.82.<br>2. Intervention group<br>• pre-test=0.70<br>• post-test=0.87	Double-blinded, cluster randomized controlled trial.	The skill-based approach showed a positive impact on the stigma of the health care professional.
7	**Ng YP, Rashid A, O'Brien F (2017)**	Randomized primary care nurses(n= 207) from Penang, Malaysia with Video-Based Contact Intervention(VBCI).	1. Baseline score= 40.<br>2. Post-intervention score= 34.	Malay version= 0.76.	Malay version.	Brief VBCI is effective in improving stigma attitudes in primary care nurses.
8	**Destrebecq, A., et al. (2018)**	Multicentric, observational, cross-sectional study on students of nursing, physiotherapy, occupational therapy, and dietetics from the university of Milan, Italy(n=561).	Not reported.	Subscales of 15 item OMS-HC are 0.76, 0.83 and 0.82.	An Italian version of 12 and 15 item OMS-HC been used.<br>The aim of the study was to examine the psychometric characteristics of the Italian version scale.	The Italian version of the OMS-HC reported valid and reliable. Older aged students showed lower stigma score.
9	**van der Maas, M. et al., (2018)**	Staff from 6 Community Health Centers(CHC) from Toronto, Canada(n=190).	Total mean score= 30.38	Sample= 0.766	Factor analysis showed good absolute. Comparing different scales designed to measure stigma were included to assess the external validity of 15-item OMS-HC.	15-item OMS-HC appropriate to be used in CHC settings and advantageous to others scales.

No.	Study	Participants	Full score	Cronbach's alphas	Remarks	Conclusions
**10**	**Laraib A, et al., (2018)**	3rd-year medical students, MBBS qualified teachers, trainee medical officers, house officers and clinicians from 5 major hospitals along with teaching institute from Abbottabad, Pakistan(n=553).	Not reported.	Not reported.	Psychiatrists, 1st and 2nd-year medical students were excluded.	Stigma does exist among healthcare providers.<br>The attitudes sub-scale reported to be high while the sub-scales on help seeking and disclosure and social distance were lower.
**11**	**Happell, B., et al. (2019)**	Nursing undergraduate students from universities located in North−Western Europe and Australia. (n=423).	Not reported.	Between 0.6-0.8 similar to study no.1 Modgill G, et al., (2014).	• Part of ongoing research on nurse education curricula (The Commune Project).<br>• The aim of the study was to assess the validity of OMS-HC through confirmatory factor analysis and Rasch modeling.<br>• Final version retains 13 items of 15 items OMS-HC.<br>• Only conducted on nursing students.	OMS-HC should not be a global estimate of stigma but could help in evaluating the efficacy of an anti-stigma intervention.
**12**	**Douglass M, Moy B(2019)**	• P3 pharmacy students from Northeastern University, Boston, Massachusetts in USA(n=93).<br>• A 90-minute intervention composed of three activities to generate discussion on the stigma of mental illness using Buzzfeed video, a Reddit discussion thread, and an AsapSCIENCE video.	• Pre-intervention= 36.8.<br>• post-intervention= 35.1.	Not reported.	Majorities reported that pharmacists should have an active role in reducing stigma.	Stigma significantly decreased according to the attitudes and disclosure/help-seeking subcategories, but not the social distancing subcategory.

No.	Study	Participants	Full score	Cronbach's alphas	Remarks	Conclusions
**13**	**Mötteli, S., et al., (2019)**	A survey on clinical staff members from 15 wards from the Psychiatric University Hospital Zurich during September and October 2017(n=110).	Mean=2.00=Sum=30	Total scale=0.75. For subscales of attitude=0.60, for disclosure and help-seeking=0.48 and for social distance=0.78.	• First research to investigate the relationship of improved recovery attitude with stigma in healthcare providers.<br>• A German version of 15-OMS-HC and Recovery Attitude Questionnaire -7 scale been used.<br>• All data were anonymously collected and ethical approval was not required.<br>• A low response rate of 36.0%.	• Participants with more optimistic recovery attitude were less stigmatized towards people with mental illness.<br>• To improve the anti-stigma campaign against the stigma of mental illness, more emphasis should be on the recovery attitudes.
