# Supplementary material for: Association between attitudes of stigma toward mental illness and attitudes toward adoption of evidence-based practice within health care providers in Bahrain
Source: PLoS One. 2019 Dec 2;14(12):e0225738. doi: 10.1371/journal.pone.0225738 (PMC6886841; doi:10.1371/journal.pone.0225738)
Supplement: Attachment — Submitted filename: Response to Reviewers.pdf [file pone.0225738.s003.pdf]

Dear Prof. Dr. Kamolz and respectable reviewers,

Hope this finds you well.

We would like first to thank you for your review and comments on our manuscript.

Reviewer 1: We reduced the introduction, material, and methods (especially the measures section). Standard English and the citations were corrected using professional service “Editage”. Thank you for your comments and suggestions. They were very helpful.

Reviewer 2:

1. The introduction was modified according to the suggestions provided.
2. Language usage, spelling, and grammar editing service of the resubmitted manuscript was provided by a professional service “Editage”.
3. An Arabic version requires validation and it would not be applicable for those who are non-Arabic speakers healthcare providers in Bahrain. Our sample would be limited to locals and Arabs. English, on the other hand, is an official language in the country and the Bahraini healthcare system. We provided our contacts and ethical code in the questionnaire. We responded to all inquiries professionally and we assume that participants did understand the questions and respond correctly.
4. We added a response to this point in the manuscript (Measures). We chose to use the standardized 15-item version, as recommended in a study which examined the scale’s properties (Modgill, Patten et al. 2014).This scale was reduced to 15-items because of weak item-total correlation (below 0.20) found in four items and additional item was dropped after cross-loaded across all three factors equally; ending with the 15-item version.
5. A regression model was added in the manuscript with a new Table that summaries the findings.

All healthcare providers (whether Bahrainis or non-Bahrainis) must pass English licensing exams and are communicating with co-workers or English speaking patients through English. In Bahrain, all Medical/Nursing universities, training programs for healthcare providers use English as the official language. The majorities are locals who are bilingual (Arabic, English) but (37.4%) as been reported in our sample are English speaking Expats (mainly from India, Philippines, and other countries). We agree with your point that was mentioned already in the ms(limitations section) that

our sample has the potential for sampling bias, oversampling the females and nurses based on their high response and reachable participation. We added a further regression analysis for the data for your kind information, and out of the journal restrain further analysis of the data would be out of the scope of our aim of the study. Thank you for your comments and suggestions. They were very helpful.

Bibliography

Modgill, G., S. B. Patten, S. Knaak, A. Kassam and A. C. H. Szeto (2014). "Opening minds stigma scale for health care providers (OMS-HC): examination of psychometric properties and responsiveness." BMC psychiatry **14**(1): 120.
