# Supplementary material for: Association between attitudes of stigma toward mental illness and attitudes toward adoption of evidence-based practice within health care providers in Bahrain
Source: PLoS One. 2019 Dec 2;14(12):e0225738. doi: 10.1371/journal.pone.0225738 (PMC6886841; doi:10.1371/journal.pone.0225738)
Supplement: Attachment — Submitted filename: PONE-D-19-17947.docx [file pone.0225738.s002.docx]

The question of attitudes of health care providers in Bahrain towards people with mental illness and toward adoption of evidence-based practice is an important one and I commend the authors for attempting to address it. The sample size, a total of 547 health care providers, is a real strength of this study. However, the following major and minor points limit my enthusiasm for this ms.

1. The introduction needs to be more focussed and tailored towards the aims of the study. While it is interesting to read about the etymology of stigma, for example, it does not add much to the understanding to the stigma individuals with mental illness encounter in Bahrain. It would be more important to focus on why the authors decided to carry out the research more explicitly and discuss research that has been carried in Bahrain.
2. The English language of the manuscript needs work. There are a lot of grammatical errors in the manuscript, which should therefore be checked by a native speaker.
3. With that in mind I wonder why the researchers did not use an Arabic translation of the questionnaires. How did they assure that participants understand all of the questions correctly?
4. Why was the OMS-HC scale shortened to 15 items? The ms seems to suggest this is common but doesn’t state this explicitly.
5. The authors use t-test to compare a lot of groups. More advanced statistical methods would be advisable. Moreover, how was multiple testing taken into account?
